# Differential *H. pylori*-Induced MAPK Responses Regulate Lewis Antigen Expression and Colonization Density on Gastric Epithelial Cells Between Children and Adults

**DOI:** 10.3389/fimmu.2022.849512

**Published:** 2022-03-08

**Authors:** Yao-Jong Yang, Chia-Ling Lu, Bor-Shyang Sheu

**Affiliations:** ^1^ Departments of Pediatrics, National Cheng Kung University Hospital, College of Medicine, National Cheng Kung University, Tainan, Taiwan; ^2^ Institute of Clinical Medicine, National Cheng Kung University Hospital, College of Medicine, National Cheng Kung University, Tainan, Taiwan; ^3^ Department of Internal Medicine, National Cheng Kung University Hospital, College of Medicine, National Cheng Kung University, Tainan, Taiwan

**Keywords:** *H. pylori*, colonization, Lewis antigen, MAPK, age

## Abstract

*Helicobacter pylori* causes gastrointestinal diseases, the manifestations of diseases are more serious in adults than in children. Lewis antigen expressions on the gastric epithelium serves as receptors targeted by *H. pylori*. Moreover, the MAPK signaling pathway involves glycoprotein synthesis of Lewis antigens. We aimed to investigate whether differences in *H. pylori*-induced MAPK responses mediate gastric Lewis antigens expression and colonization density differently in children and adults. We used human stomach fetal epithelium (HSFE) and SV40-immortalized human normal gastric epithelial (GES-1) cell lines to mimic primary gastric epithelium of children and adults, respectively. *H. pylori* colonization intensity and Lewis antigens were significantly higher in GES-1 than in HSFE cells, whereas IL-8 and IL-6 levels were significantly higher in HSFE than in GES-1 cells after infection. c-Jun N-terminal kinase (JNK) siRNA and inhibitor (SP600125) experiments showed that Lewis antigen expression and *H. pylori* colonization were reduced in GES-1 cells but increased in HSFE cells. Furthermore, p-p38 intensity was significantly higher in the superficial epithelium of the children than in the adults with/without *H. pylori* infection. The overexpression of p38 in GES-1 cells downregulated *H. pylori*-induced JNK activity mimicking *H. pylori* infection in children. In conclusion, a higher p38 expression in gastric epithelium counteracting JNK activity in children may contribute to lower Lewis antigen expression and colonization density than in adults after *H. pylori* infection.

## Introduction

*Helicobacter pylori* infection causes chronic gastritis, peptic ulcer disease, and adenocarcinoma in humans (1, 2). The cross-talk of bacteria and host immunity mediates the pathogenesis of subsequent disease (3), and the adhesion and colonization of *H. pylori* to the gastric epithelium is the key step in establishing the infectious process (4, 5). Adhesion molecules such as BabA and SabA proteins are produced by *H. pylori* and have been shown to adhere to the glycan-rich domains of the gastric epithelium (5–7). The BabA protein recognizes both H-type 1 and Lewis b (Le^b^) antigens expressed on gastric mucosa leading to the initial step of infection (5). Subsequently, SabA adhesin mediates *H. pylori* binding to inflamed gastric mucosa by recognizing sialyl-Lewis a (sLe^a^) and sialyl-Lewis x (sLe^x^) antigens to establish persistent colonization (6). Therefore, factors that can regulate the intensity of Lewis antigens are responsible for *H. pylori* colonization density on the gastric epithelium.

Acquisition of *H. pylori* infection occurs mainly in early childhood. Several competitive studies have shown different immune responses between children and adults with *H. pylori* infection (8–12). In addition, the bacterial load and presence of virulence factors have been shown to play major roles in the differences in *H. pylori*-associated clinical diseases between children and adults (13). In our previous study, we found that *H. pylori*-infected adults had a significantly higher bacterial density and Le^b^ intensity over the corpus than *H. pylori*-infected children (14). In addition, Nogueira et al. reported that human gastric Le^b^ antigen expression was associated with increased age (15). We also previously found that the gastric sLe^x^ expression in mice was age dependent (16). However, little is known about differences in the acute responses of cytokine production, Lewis antigen expression, and subsequent colonization density related to mitogen-activated protein kinase (MAPK) signaling on gastric epithelium between children and adults.

*H. pylori* has been shown to activate extracellular signal-regulated kinases (ERK), p38, and c-Jun N-terminal kinase (JNK) MAPKs in gastric epithelial cells (17). Kim et al. reported that *H. pylori* induces p38 MAPK activation, which may reduce mucin synthesis and apoptosis on gastric epithelial cells (18). Another study demonstrated that p38 and ERK MAPK are involved in reducing mucin synthesis, which is induced by the lipopolysaccharide of *H. pylori* (19). It is therefore reasonable to hypothesize that the differences in infectious consequences between children and adults are mediated by differences in MAPK activation and Lewis antigen synthesis after *H. pylori* infection. The aim of this study was to investigate differences in the MAPK-Lewis antigen-adhesion pathway between mature and immature gastric epithelium.

## Methods

### Cells and Culture

Human gastric epithelial immortalized GES-1 cells representing the mature (adult) gastric epithelium (a gift from Prof. Jia, Shandong University, China) were cultured in DMEM medium (Corning, Tewksbury, MA, USA) supplemented with 10% FBS with 5% CO_2_ at 37°C. The cells were subcultured every second day. Prior to the bacterial infection study, the cells were incubated in FBS‐free DMEM medium overnight at 37°C in 5% CO_2_. Human fetal stomach cells (HFSC, Innoprot, USA) were purchased from Smartec Scientific Corp., Taiwan. The HFSC cells were cultured in Epithelial Pro-Conditioned Cellutions Medium (D‐PRO‐015, DV Biologics, CA) with 5% CO_2_ at 37°C. The medium was changed every second day, and the cells were subcultured before they overgrew until they were induced to human stomach fetal epithelium (HSFE) cells, a cell line to represent immature (child) gastric epithelium. Before the study conduction, the cells were incubated in D‐PRO medium and DMEM (1:1 mix) with 10% FBS. Prior to the bacterial infection study, the cells were starved in FBS‐free DMEM medium overnight at 37°C in 5% CO_2_.

### Bacterial Culture Method and Infection

*H. pylori* strain HP238 isolated from a clinical patient was used, which has been shown to express CagA, VacA, and BabA proteins in previous studies (20). The bacteria were maintained on CDC blood agar (BBL, Microbiology Systems, Cockeysville, MD) or in Brucella broth containing 10% horse serum (Gibco BRL, Life Technologies, Rockville, MD) at 37°C and incubated under micro‐aerophilic conditions (10% CO_2_, 5% O_2_ and 85% N_2_) for 24-48 hours. The bacteria were transferred to phosphate-buffered saline (PBS) before infecting the cells. Growth density was measured spectrophotometrically at 600 nm. The infectious dose of bacteria was 1×10^8^ bacteria/ml at an OD of 1. Cells were infected at a multiplicity of infection (MOI) of 100:1.

GES-1 and HSFE cells (1 x 10^6^/well) were grown in 6-well culture dishes until approximately 80% confluence. After washing with PBS, *H. pylori* (HP238, MOI 100) was added to the wells without centrifugation and incubated for 30 minutes to 8 hours. The culture supernatant of each well was collected and stored for further use.

### Inhibitors and siRNA Transfection Target for MAPK Activity

Cells were pretreated with pharmacological MAPK inhibitors for p38 (SB203580), JNK (SP600125), and ERK (PD98059) (all from Cell Signaling Technology) with variable concentrations, and DMSO as a negative control for 1 hour before *H. pylori* infection. siRNA against p38, ERK, JNK and control siRNA (scrambled siRNA, all from Life Technologies, Thermo Fisher Scientific, Carlsbad, CA, USA) (**Table 1** **)** were transfected into the GES-1 and HSFE cells, respectively, with Lipofectamine RNAi MAX according to the manufacturer’s instructions.

**Table 1:** The siRNA sequence of MAPK for transfection.

Target	Name	Sequence (5’ > 3’)
ERK-1	MAPK2	GGAAAAGCUCAAAGAACUAtt
ERK-2	MAPK1	CCGGAUGUUAACCUUUAACtt
JNK-1	MAPK8	GGGAUUUGUUAUCCAAAAUtt
JNK-2	MAPK9	GGGAUUGUUUGUGCUGCAUtt
p38	MAPK14	GGAAUUCAAUGAUGAUGUGUAUtt
Negative control	Negative Control #1	siRNA has no significant sequence similarity to mouse, rat, or human gene sequences.

### Flow Cytometry for Bacterial Adhesion and Receptor Expression Assay

After *H. pylori* infection, each well with GES-1 and HSFE cells was washed three times with PBS to remove unbound bacteria. The HSFE and GES‐1 cells were detached by 0.2% EDTA at 37°C for 5 minutes, then fixed with 70% alcohol at 4°C. The cells were incubated with each primary antibody (B0471: anti-*H. pylori*, Dako; 2-25LE: anti-Lewis b, abcan; CD15s: anti-sialyl‐Lewis x, BD) at 4°C for 30 minutes, and then incubated with the secondary antibody Alexa Fluor 488, 555 (abcan, Invitrogen) at 4°C for 30 minutes. After washing with DPBS, fluorescence was measured using BD FACSCalibur (Cell Quest). The percentage or mean fluorescence intensity was measured using FlowJo v10 (Genetech Biotech Co. Ltd. Tw). In brief, observation of adhesion intensity for *H. pylori* was based on flow detection of one cell at a time to measure its anti-*H. pylori* fluorescence value. We used infectious dose MOI 100 in each test and assumed the mean intensity could be represented the bacterial adhesion amount. All tests were done in triplicate.

### Enzyme-Linked Immunosorbent Assay (ELISA) for Cellular Cytokine Expression

The culture supernatants were centrifuged at 35,000 rpm for 5 minutes to remove bacteria and cell debris, and then stored at -80°C until use. The concentrations of IL-6 and IL-8 were measured at 0-4 hours by ELISA (R & D System) according to the manufacturer’s instructions. The absorbance of each micro-plate was read on a spectrophotometer using 450 nm as the primary wavelength and 570 nm as the reference.

### Western Blotting to Detect MAPK Phosphorylation

GES-1 and HSFE cells were cocultured with *H. pylori* for the appropriate times (0.5-8 hours). Total protein of the cells was lysed using RIPA (Millipore), and the protein concentration was quantified using a BCATM Protein Assay Kit (PIERCE). Samples containing equal amounts of total protein were subjected to SDS-PAGE (Bio-Rad system). Proteins were transferred to a 0.25-μm Transblot nitrocellulose membranes (Bio-Rad), and the membranes were blocked using 5% (w/v) milk in TBST. Immunodetection of phosphorylated or total MAPKs was performed by incubating the membranes with anti-p-p38, anti-p-ERK1/2, anti-p-JNK, anti-p38, anti-ERK, and anti-JNK primary antibodies (all from Cell Signaling Technology) at a dilution of 1/1000, except for anti-actin antibody (Millipore) which was used at a dilution of 1/2000 in 5% (w/v) milk in TBST. Secondary goat anti-rabbit and anti-mouse (Chemicon/Millipore) antibodies were used at a dilution of 1/10000 in 5% milk in TBST. Western blots were developed using ECL detection reagent (Millipore) and exposed to Amersham HyperfilmTM MP (GE Healthcare). The quantification of MAPK activity was measured by densitometry from 0.5 to 4 hours.

### Plasmid DNA Transfection to Generate GES-1 Cells Overexpressing p38

The p38 (CRK) (NM_016823) Human Tagged ORF Clone was purchased from OriGene Technologies Inc. (Rockville, MD, USA). The procedure for generating GES-1 cells overexpressing p38 was conducted following the manufacturer’s instructions. In brief, the TransIT-X2 was warmed to room temperature and gently vortexed before use. A total of 1.5 ml serum-free culture medium was added to a sterile test tube, followed by the addition of 15 μl plasma DNA (1 μg/μl) and gentle pipetting to mix thoroughly. Next, 45 μl TransIT-X2 was added to the diluted DNA mixture and mixed thoroughly. The mixture was incubated at room temperature for 15 to 30 minutes, and then drops of the TransIT-X2/DNA complex were added to each area of the dish, with gentle shaking to allow for even distribution. The dishes were then incubated for 24-72 hours. The cells were collected for tests *in vitro*.

### Immunohistochemical Staining and Scoring for Gastric MAPK Activity

Patients with dyspepsia receiving esophagogastroduodenoscopy examination with biopsies for *H. pylori* infection were enrolled. Topographical specimens from the gastric antrum, body and cardia of each *H. pylori*-infected or non-infected child and adult (each group n=4) were used to analyze the expression of MAPK activity, which was determined by immunohistochemical staining. We selected only for patients with chronic gastritis and excluded who had peptic ulcers or gastric cancers. The age and sex were matched between *H. pylori*-infected and non-infected children and adults. Tissue sections were treated with primary antibodies against p-p38 (1:100), p-JNK (1:400), and p-ERK (1:500) (all from Cell Signaling Technology) at 4°C overnight in a humidified chamber. Setting step followed the manufacturer’s instructions (ECL System, Millipore Corporation, USA). Specifically bound peroxidase was detected by Chemiluminescent HRP Substrate (Max Polymer Detection System, NovolinkTM), and then exposed to X-rays (GE Healthcare, UK) for an optimal duration.

MAPK expression was analyzed in SE cells, DG cells, and LP mononuclear cells and stromal cells. The intensity of each MAPK expression was scored (range 0–4) according to the distribution of positively stained SE cells, DG cells, and LP mononuclear cells and stromal cells. The quantitative score of each MAPK expression on the topographic portion was as follows: score 0, negative or less than 1% of the stained cells; score 1, 1% to 25% of the stained cells; score 2, 25% to 50% of the stained cells; score 3, 50% to 75% of the stained cells; and score 4, 75% to 100% of the stained cells (**Supplemental Figure 1**).

### Statistics and Figure

The statistics and figure were performed by software Prism6. The Student’s t-test was applied as appropriate for the parametric differences. The differences were considered to be significant at P<0.05.

## Results

### Induction of Human Stomach Fetal Epithelium (HSFE) Cells

**Figure 1:**
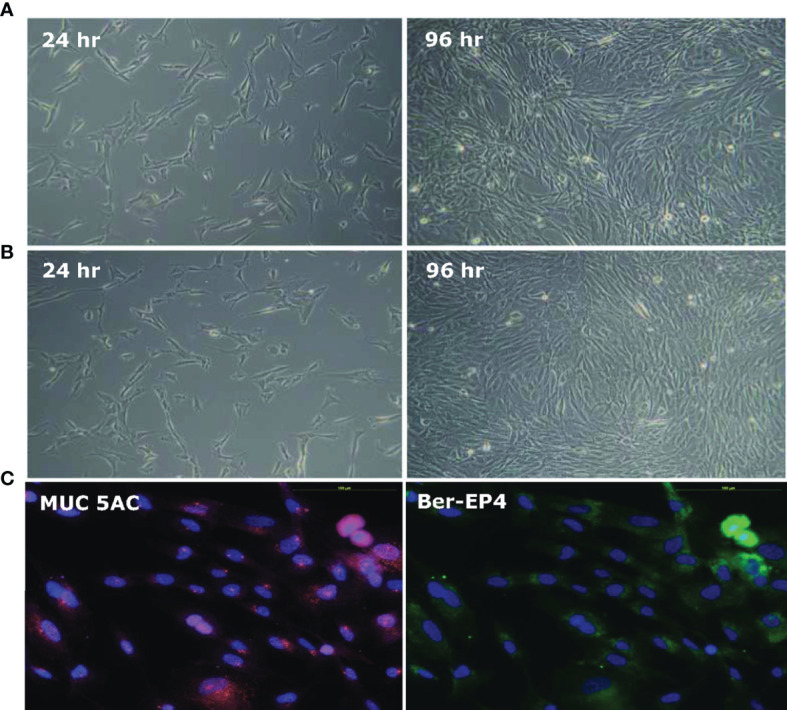
Establishment of human stomach fetal epithelium (HSFE) cells from human fetal stomach cells (HFSC). Human fetal stomach cells (HFSC) were cultured in **(A)** D‐PRO‐015 and **(B)** modified 2/3 E-Pro and 1/3 DMEM Medium with 5% CO2 at 37°C for 24 and 96 hours under a 10X phase contrast microscope. The D‐PRO medium was changed every second day, and the cells were subcultured before they overgrew until they were induced to HSFE cells. **(C)** Most of the cells were positive for anti-MUC5AC (pink) monoclonal antibodies, a marker of gastric epithelial cells, with Ber-EP4 (blue) nuclear staining and negative for vimentin (not shown).

HSFE cells were induced using Epithelial Pro‐Conditioned Cellutions Medium from human fetal stomach cells (HFSC) according to the manufacturer’s instructions (DV Biologics, Costa Mesa, CA). **Figure 1** shows the primary cultures of HFSC cells after seeding under a phase contrast microscope at 10X at 24 and 96 hours. The cell morphology was characterized by an elongated cytoplasm and stratification with D Pro-Conditioned Medium (**Figure 1A**) and modified 2/3 E-Pro and 1/3 DMEM Medium (**Figure 1B**). Most of the cells were positive for anti-MUC5AC (pink) monoclonal antibodies, a marker of gastric epithelial cells, with Ber-EP4 (blue) nuclear staining (**Figure 1C**) and negative for vimentin (data not shown).

### The *H. pylori* Colonization Rates Were Significantly Higher in the GES-1 Cells Than in the HSFE Cells

**Figure 2:**
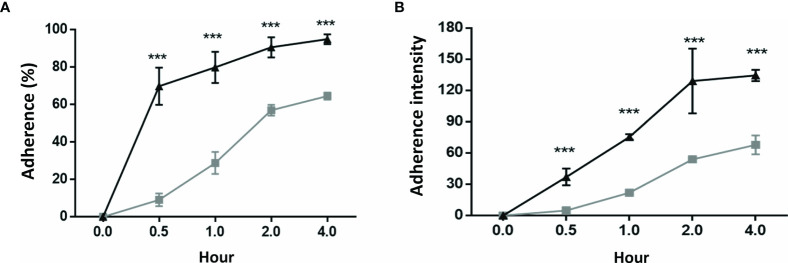
*H. pylori* colonization rate and intensity between GES-1 and HSFE cells over time. GES-1 (black triangles) and HSFE (gray squares) cells were challenged with *H. pylori* (MOI 100) for 0, 0.5, 1, 2, and 4 hours. The cells were washed thrice with PBS to remove unadhered *H. pylori.* The adherent rates of *H. pylori* **(A)** percentage and **(B)** intensity were detected using specific antibodies by flow cytometry. Data are expressed as means ± SD (in triplicate). Statistical analysis was performed for each measurement with comparisons with the HSFE cells at each time period (***P < 0.001).

Comparisons of the cell colonization rate and bacterial density between the GES-1 and HSFE cells after *H. pylori* challenge at various time periods are shown in **Figure 2**. The results showed that both bacterial colonization rate and density were positively correlated with incubation period. Moreover, both *H. pylori* colonization rate (**Figure 2A**) and density (**Figure 2B**) were significantly higher in the GES-1 cells than in the HSFE cells (P < 0.001).

### Higher Lewis b and Sialyl-Lewis x Antigen Expressions in the GES-1 Cells Than in the HSFE Cells After *H. pylori* Challenge

**Figure 3:**
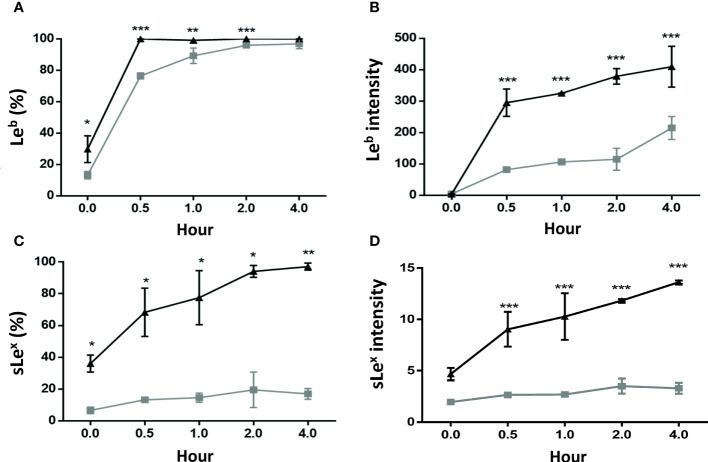
Lewis b (Le^b^) and sialyl-Lewis x (sLe^x^) antigen expressions after *H. pylori* infection between GES-1 and HSFE cells over time. GES-1 (black triangles) and HSFE (gray squares) cells were challenged with *H. pylori* (MOI 100) for 0, 0.5, 1, 2, and 4 hours. The cells were washed thrice with PBS to remove unadhered *H. pylori*. The percentage and intensity of Le^b^ **(A, B)** and sLe^x^ **(C, D)** expressions were detected using specific antibodies by flow cytometry. Data are expressed as means ± SD (in triplicate). Statistical analysis was performed for each measurement with comparisons with the HSFE cells at each time period (*P<0.05, **P < 0.01, ***P < 0.001).

**Figure 3** shows Lewis b (Le^b^) and sialyl-Lewis x (sLe^x^) antigen expressions after *H. pylori* challenge in the GES-1 and HSFE cells at 0-4 hours over time. *H. pylori* infection induced Le^b^ and sLe^x^ antigen expressions in both GES-1 and HSFE cells in a time-dependent manner (**Figures 3A–D**). The percentage of Le^b^ antigen expression in the GES-1 cells rapidly increased to a full expression (100%) after *H. pylori* challenge, however this was slower in the HSFE cells (**Figure 3A**). Moreover, the intensity of Le^b^ antigen expression after challenge was significantly higher in the GES-1 cells than in the HSFE cells at each time period (**Figure 3B**). In addition, *H. pylori* induced a time-dependent increases in the percentage and intensity of sLe^x^ antigen expression in the GES-1 cells. However, the sLe^x^ antigen expression after challenge was significantly lower in the HSFE cells than in the GES-1 cells **(** **Figures 3C, D** **)**.

### Differential Cytokine and MAPK Induction Between the GES-1 and HSFE Cells by *H. pylori*

**Figure 4:**
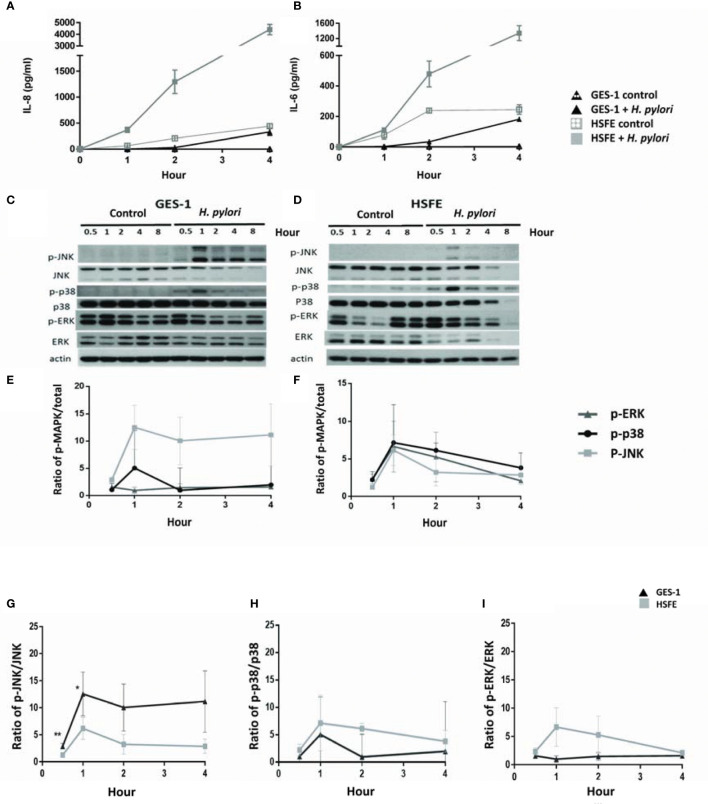
Differences in *H. pylori*-induced cytokine expressions and phosphorylation of MAPK signaling between GES-1 and HSFE cells over time. GES-1 (black triangles) and HSFE (gray squares) cells were infected (solid) or not infected (hollow) with *H. pylori* (MOI 100) for 0, 0.5, 1, 2, and 4 hours. The levels of IL-8 **(A)** and IL-6 **(B)** were detected by ELISA. The phosphorylation of JNK, p-38, and ERK in GES-1 **(C)** and HSFE **(D)** cells after *H. pylori* challenging were measured by Western blot and were quantitated in **(E, F)**, respectively. For comparisons of the differential MAPK activation between GES-1 and HSFE cells after *H. pylori* infection, the ratios of **(G)** p-JNK/JNK, **(H)** p-p-38/p-38, and **(I)** p-ERK/ERK are shown between GES-1 and HSFE cells. (*P < 0.05, **P < 0.01).

*H. pylori* infection induced the expressions of IL-8 and IL-6 cytokines in both the GES-1 and HSFE cells (**Figures 4A, B** **)**. Interestingly, the cytokine levels induced by *H. pylori* were much higher in the HSFE cells than in the GES-1 cells. In addition, the Western blot results showed that *H. pylori*-induced MAPK phosphorylation activity reached the highest level at 1 hour, and that the induction levels were different between the GES-1 and HSFE cells (**Figures 4C, D** **)**. In the GES-1 cells, *H. pylori* induced a persistently high p-JNK/JNK ratio, however the p-p38/p38 ratio was only transiently activated at 1 hour. The phosphorylation of ERK was not obviously induced (**Figure 4E**). However, in the HSFE cells, *H. pylori* activated MAPK activity at 1 hour, and then the levels decreased (**Figure 4F**). Comparing MAPK activity between the GES-1 and HSFE cells, *H. pylori* induced a significantly higher p-JNK/JNK ratio in the GES-1 cells than in the HSFE cells (**Figure 4G**). However, *H. pylori* induced higher p-p38/p38 (**Figure 4H**) and p-ERK/ERK (**Figure 4I**) ratios in the HSFE cells than in the GES-1 cells.

### MAPK Activity Impacted the Intensity of *H. pylori* Colonization Differently in the GES-1 and HSFE Cells

**Figure 5:**
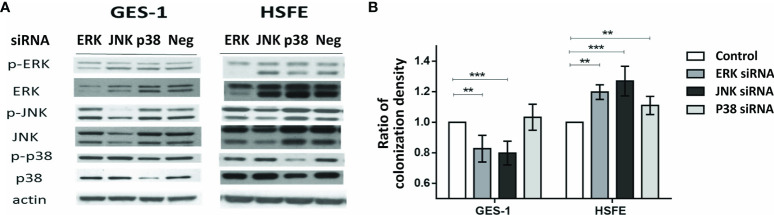
The impact of MAPK activation after *H. pylori* challenge on the colonization intensity between GES-1 and HSFE cells. GES-1 and HSFE cells were transfected with ERK, JNK, p38, and negative control (Neg) siRNA 10 ρg with 8 μl RNAi MAX Lipofectamine. The cells were then challenged with *H. pylori* (MOI 100) for 1 hour. **(A)** SiRNA transfection efficiency was confirmed by Western blot. **(B)** The intensity of *H. pylori* colonization was detected by flow cytometry using specific antibodies. Each test was in triplicate. Data are expressed as means ± SD. Statistical analysis was performed for each measurement with comparisons to the negative control. **P < 0.01, ***P < 0.001).

We then evaluated whether *H. pylori* colonization intensity was associated with the differential phosphorylation activity of MAPKs between the GES-1 and HSFE cells. **Figure 5** shows the impact of MAPK activation after *H. pylori* challenge on the colonization intensity in the GES-1 and HSFE cells. The GES-1 and HSFE cells were transfected with 10 ρg siRNA of p38, JNK, and ERK, with 8 μl RNAi MAX Lipofectamine. The siRNA transfection efficiency was confirmed by Western blot (**Figure 5A**). In the GES-1 cells, knockdown of JNK and ERK activity significantly impaired the *H. pylori* colonization density. However, p38 knockdown in the GES-1 did not affect *H. pylori* colonization density. In contrast to the GES-1 cells, knockdown of ERK, JNK, and p38 activity significantly increased *H. pylori* colonization intensity in the HSFE cells (**Figure 5B**).

### p-JNK Activity Affected the Intensity of Gastric Lewis Antigen Expression

**Figure 6:**
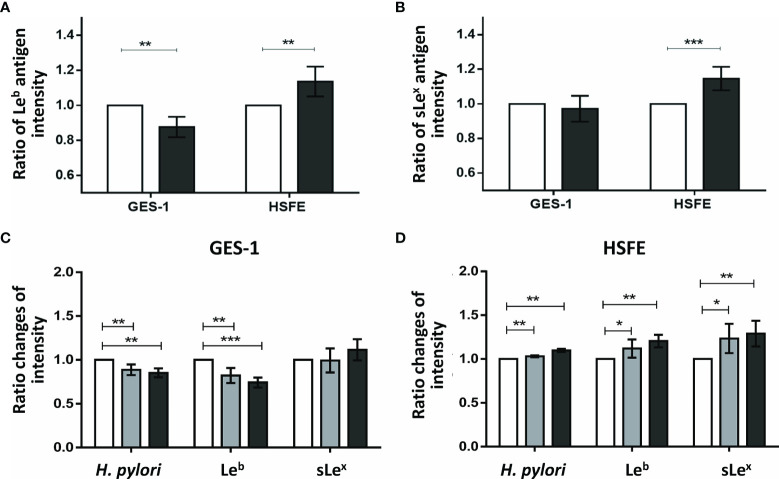
The impact of p-JNK activity on the intensity of Lewis antigen expression in GES-1 and HSFE cells. GES-1 and HSFE cells were transfected with JNK (black) and negative control (white) siRNA 10 ρg with 8 μl RNAi MAX Lipofectamine. The cells were challenged with *H. pylori* (MOI 100) for 1 hour. The intensity of **(A)** Le^b^ and **(B)** sLe^x^ antigen expressions were detected using specific antibodies by flow cytometry. Pretreatment with a p-JNK inhibitor in **(C)** GES-1 and **(D)** HSFE cells at different doses (0, white; 12.5 μM, gray; 25 μM, black) for 1 hour. The cells then were co-cultured with *H. pylori* (MOI 100) for 1 hour. The intensity of *H. pylori* colonization, Le^b^, and sLe^x^ antigen expressions were detected using specific antibodies by flow cytometry. Data are expressed as means ± SD (in triplicate). Statistical analysis was performed with each measurement with comparisons to the negative control. (*P < 0.05, **P < 0.01, ***P < 0.001).

As p-JNK activity was shown to have the greatest impact on the difference in *H. pylori* colonization density between the GES-1 and HSFE cells, we further investigated whether p-JNK activity affected differences in Lewis antigen expressions between the GES-1 and HSFE cells using siRNA and a p-JNK inhibitor (**Figures 6A, B** **)**. Knockdown of JNK activity significantly decreased Le^b^ antigen intensity in GES-1 cells after *H. pylori* challenge but significantly increased the Le^b^ antigen intensity in HSFE cells (**Figure 6A**). However, only sLe^x^ antigen intensity was significantly upregulated in the HSFE cells after *H. pylori* challenge by JNK knockdown (**Figure 6B**). Moreover, pretreatment with a p-JNK inhibitor also decreased Le^b^ and *H. pylori* colonization intensities in GES-1 cells (**Figure 6C**) and increased the Le^b^, sLe^x^, and *H. pylori* colonization intensities in HSFE cells (**Figure 6D**). Both effects displayed a dose-dependent manner.

### Differential MAPK Activation in Gastric Biopsies After *H. pylori* Infection Between Adults and Children

**Figure 7:**
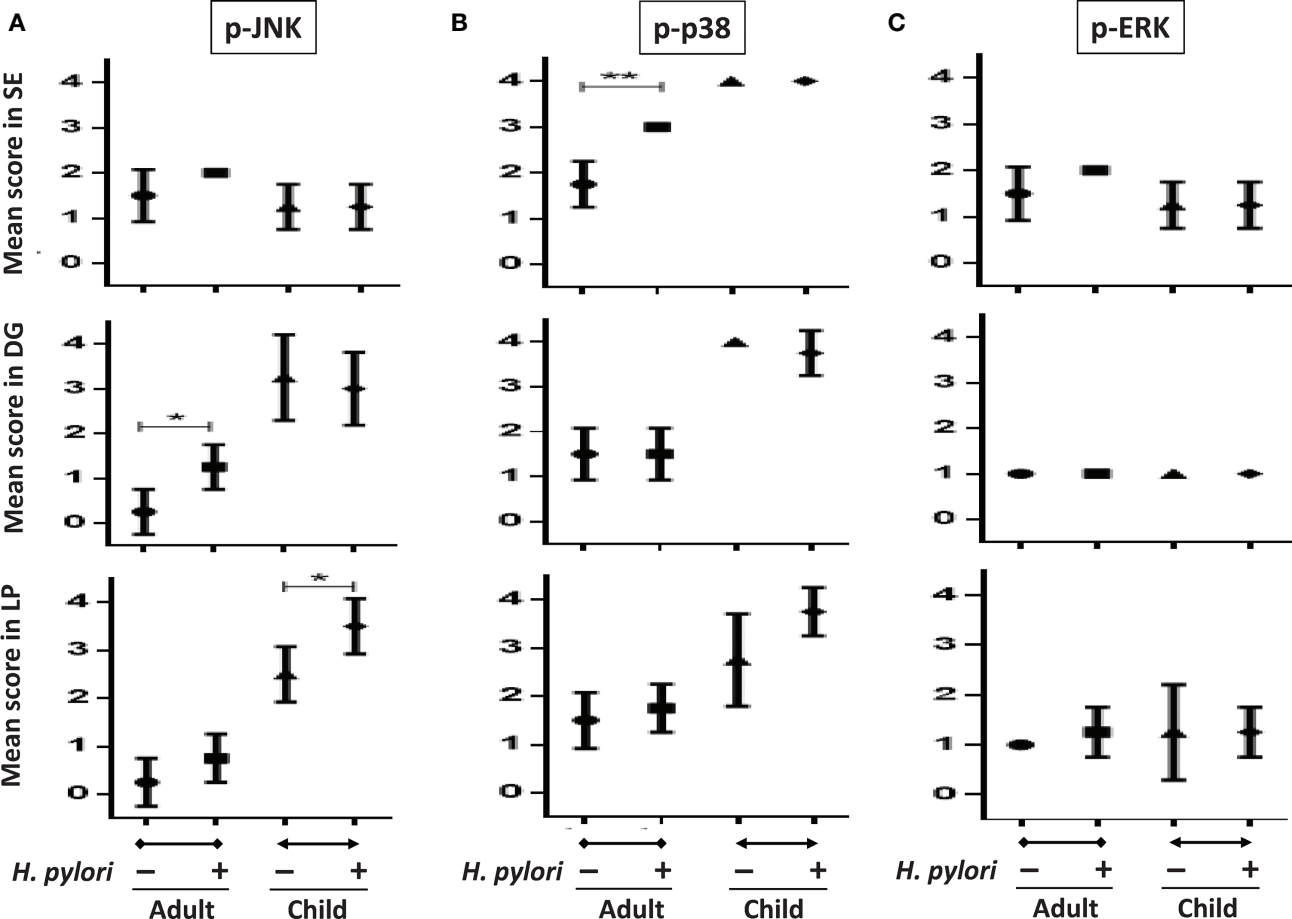
The intensity scores of p-JNK, p-p38, and p-ERK in gastric biopsy samples between children and adults with and without *H. pylori* infection. Tissue sections including topographical specimens from the antrum in adults and children with and without *H. pylori* infection (each group, n = 4), were subject to immunohistochemical staining to analyze the intensity of **(A)** p-JNK, **(B)** p-p38, and **(C)** p-ERK MAPK expressions. The sores were stratified into superficial epithelium cells (SE), deep glandular cells (DG), and lamina propria (LP) mononuclear and stromal cells in the nucleus. (*P < 0.05, **P < 0.01).

To confirm that *H. pylori* activates different gastric MAPK expressions in children and adults, we investigated the intensity of MAPK expressions in gastric biopsies using immunohistochemistry staining. **Figure 7** shows the mean intensity scores of p-JNK, p-p38, and p-ERK on gastric antrum between children and adults with and without *H. pylori* infection (each n = 4). The intensity scores were compared topographically in superficial epithelium (SE), deep glandular (DG), and lamina propria (LP) cells. The results showed that the children without *H. pylori* infection had higher antral p-p38 scores in SE, DG, and LP cells than adults without infection (**Figure 7B**). The p-JNK scores in children were also higher than those in adults in DG and LP cells. Importantly, the mean p-JNK score in the SE cells was equivalent between the children and adults (**Figure 7A**). In addition, the mean p-ERK score was not different between the adults and children topographically (**Figure 7C**). In adults with *H. pylori* infection, the mean p-JNK score was significantly higher in the DG cells compared to those without infection (**Figure 7A**). Furthermore, *H. pylori*-infected adults also had a higher p-p38 score in the SE cells than non-infected subjects (**Figure 7B**). In children, *H. pylori* infection did not significantly upregulate p-JNK, p-p38, or p-ERK scores, except for a significantly higher p-JNK score in the LP cells than in those without infection (**Figure 7A**). There were no significant differences in p-ERK scores between the adults and children with and without *H. pylori* infection (**Figure 7C**).

### Overexpression of p38 Repressed *H. pylori*-Induced p-JNK in the GES-1 Cells

**Figure 8:**
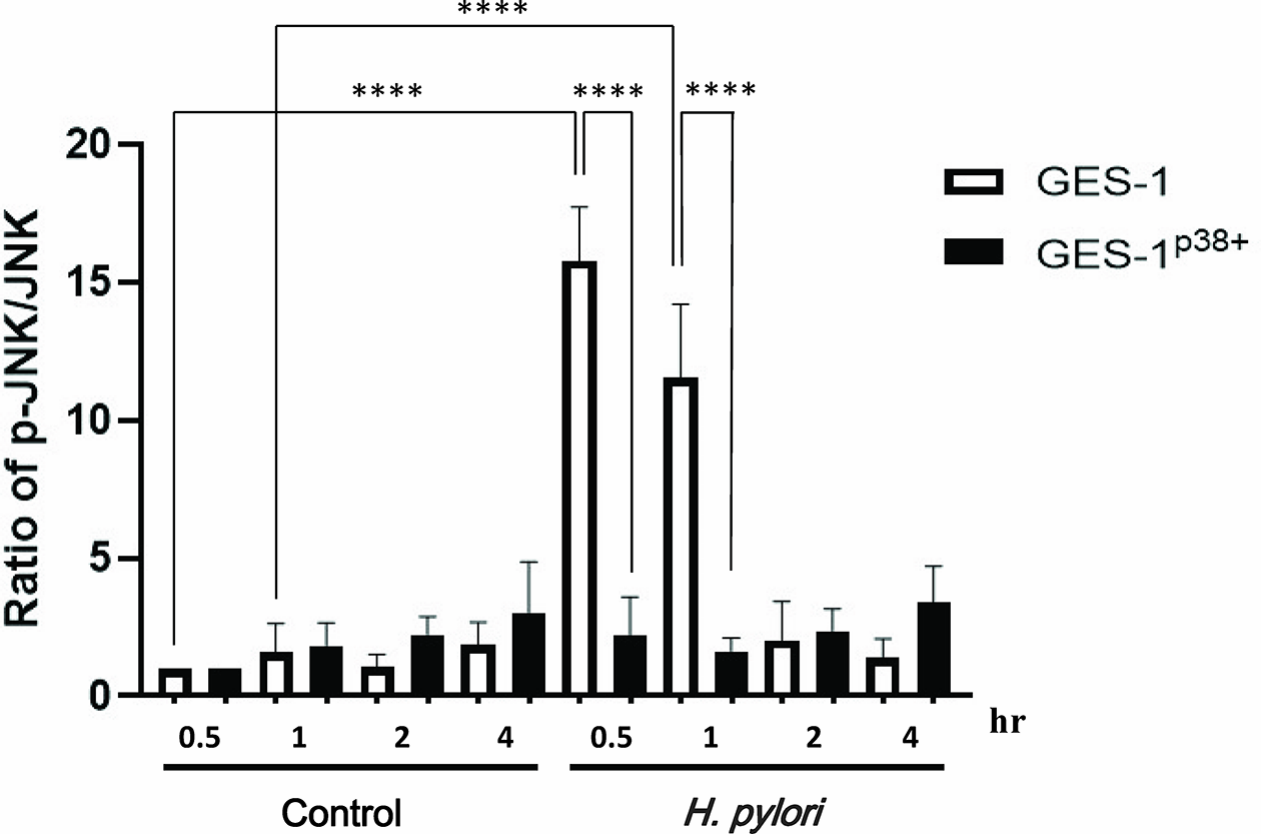
The translation levels of p-JNK, p-p38, and p-ERK in GES-1 and GES-1^p38+^ (GES-1 overexpression of p38) after *H. pylori* infection. p38 Human Tagged ORF Clone was transfected using TransIT-X2 into GES-1 cells. The TransIT-X2/DNA complex was then added to each area of the dish. The ratios of p-JNK/JNK in GES-1 and GES-1^p38+^ cells after *H. pylori* infection over time are shown. Each ratio was compared to the results at 0.5 hours in the controls. Each test was performed in triplicate. (****P < 0.0001).

As the pediatric gastric biopsies generally expressed higher p38 activity than those of the adults, and as *H. pylori* induced higher p38 phosphorylation in the HSFE cells than in the GES-1 cells, we further investigated whether the overexpression of p38 in GES-1 cells (GES-1^p38+^) could abolish *H. pylori*-induced JNK phosphorylation (**Figure 8**). The results showed that GES-1 cells infected with *H. pylori* had a significantly higher p-JNK/JNK ratio than the controls at 0.5 hours (P<0.001) and 1 hour (P<0.001). Moreover, we confirmed again that *H. pylori*-infected GES-1^p38+^ cells significantly reduced p-JNK activity at 0.5 hours (P<0.001) and 1 hour (P<0.001) compared to GES-1 cells after *H. pylori* infection (**Figure 8**). These results indicated that the higher gastric p38 activity in children counteracted the JNK phosphorylation after *H. pylori* infection, and that this may contribute to the lower gastric Lewis antigen expression and bacterial density in children than in adults with *H. pylori* infection.

## Discussion

In this study, we found that *H. pylori* induced JNK phosphorylation *in vivo* and *in vitro*, which then upregulated the expressions of gastric Lewis antigens and subsequent bacterial colonization density. The differences in p-JNK indication may explain the variety in bacterial colonization density between adults and children after *H. pylori* infection. These findings clearly show that although *H. pylori* initiates colonization in childhood, as the age increases, *H. pylori* induces vigorous JNK activity which upregulates gastric Lewis antigen expressions and higher bacterial load in the host. This is the reason why *H. pylori*-infected adults have more severe gastric inflammation and disease than children.

In this study, we used HSFE cells, which are derived from prenatal stomach tissue (HFSC) to represent immature (child) gastric epithelium. We also confirmed the property of gastric epithelial cells according to the presence of MUC5AC mucin. Consistent with clinical findings (14–16), the adult primary epithelial cell (GES-1) in the current study had a higher bacteria density than the HSFE cells after *H. pylori* infection. Moreover, the percentage and intensity of *H. pylori* load in the GES-1 cells increased rapidly than in the infected HSFE cells (**Figure 2**). Moreover, the percentage of *H. pylori* adherence was consistent with the Le^b^ and sLe^x^ expressions in both the GES-1 and HSFE cells after *H. pylori* infection (**Figure 3**). Thus, our *in vitro* study conformed again that a higher *H. pylori*-induced Lewis antigen expression in adults could contribute to the higher *H. pylori* load than in children.

Several previous studies have shown that *H. pylori* induces gastric Th1 and Th17 immune responses, and that these responses vary according to age and may determine the differences in outcomes between children and adults after *H. pylori* infection (8–12). In our previous studies, we found differences in *H. pylori*-induced Le^b^ and sLe^x^ antigen expressions between adults and children in human and animal studies (14–16). In the current study, the IL-8 and IL-6 cytokine levels induced by *H. pylori* were much higher in the HSFE cells than in the GES-1 cells. This is consistent with a previous study which reported that the gastric concentration of IL-6 was usually higher in *H. pylori*-infected children than in adults (8). In addition, Padró et al. found that the expressions of fucosyltransferases involved in the synthesis of Lewis antigens in gastric cancer cells could be specifically modulated by IL-1β and IL-6 inflammatory cytokines (21). Moreover, inflammatory cytokines have been shown to modulate the glycosylation pattern of pancreatic tumor cells, leading to increased expressions of tumor-associated sialylated antigens such as sLe^x^ (22). However, it is unclear whether the gastric inflammatory signaling pathway-related Lewis antigen induction after *H. pylori* infection is age-dependent, and whether it mediates the difference in *H. pylori* colonization density between children and adults.

*H. pylori* infection has been reported to induce MAPK activation in primary gastric cells (23). In the current study, we not only confirmed this finding but also clarified that the MAPK responses were different between infected children and adults (**Figure 4**). Both p-38 and ERK activations were higher in the HSFE cells than in the GES-1 cells after *H. pylori* infection. In contrast, *H. pylori*-infected GES-1 cells had significantly higher JNK activation than HSFE cells. Previous studies have shown that *H. pylori*-induced p38 and ERK MAPK activation can affect gastric mucin synthesis (18, 19). Therefore, we hypothesize that the higher p-38 expression in children counteracting the JNK activity after *H. pylori* infection results in lower Lewis antigen production and *H. pylori* density than in adults. We used MAPK inhibitors to investigate the effect of MAPK activation on colonization density. Interesting, JNK inhibition significantly decreased colonization density in GES-1 cells but increased colonization density in HSFE cells. Furthermore, p-38 inhibition significantly increased colonization density in HSFE cells, but did not alter colonization density in GES-1 cells (**Figure 5**). Our *in vitro* findings are the first to identify the different consequences of MAPK activation on differences in bacterial and Lewis antigen densities in children and adults after *H. pylori* infection.

In comparisons of the phosphorylation of MAPKs between gastric biopsies of children and adults, this study is the first to find higher activations of JNK and p-38 in children than in adults without *H. pylori* infection. Because both the children and adults were dyspeptic, factors other than *H. pylori-*induced MAPK activation in those without *H. pylori* infection were unknown. Consistent with the *in vitro* study, the *H. pylori*-infected adults had significantly upregulated JNK activity compared to the infected children. However, the results were different to the *in vitro* study for p-38 and ERK activation. In the transfection study, the overexpression of p38 could inhibit JNK activity in the GES-1 cells. This confirmed that a higher p38 activity in the gastric epithelium could suppress JNK expression and possibly lead to the lower Le^b^/bacterial density in children than in adults. Taken together, our results imply that JNK activation is the key factor for the differences in cytokine induction, Lewis antigen expression, and colonization load between children and adults after *H. pylori* infection.

In conclusion, our findings show that differences in the *H. pylori*-induced MAPK signaling pathway, which regulates Lewis antigen expression and bacterial density, may be responsible for the differences in clinical outcomes between children and adults.

## Data Availability Statement

The original contributions presented in the study are included in the article/**Supplementary Material**. Further inquiries can be directed to the corresponding author.

## Author Contributions

Y-JY was involved in the design and conduction of the study, interpretation of data, and in drafting the manuscript. C-LL was involved in conducting the experiments, analysis of data, and discussion of the manuscript. B-SS was involved in the setting of the study design and interpretation of data, editing and final approval of the manuscript. All authors read and approved the final version of the manuscript.

## Funding

This study was supported by a grant from the National Cheng Kung University Hospital (NCKUH-10204006), Taiwan.

## Conflict of Interest

The authors declare that the research was conducted in the absence of any commercial or financial relationships that could be construed as a potential conflict of interest.

## Publisher’s Note

All claims expressed in this article are solely those of the authors and do not necessarily represent those of their affiliated organizations, or those of the publisher, the editors and the reviewers. Any product that may be evaluated in this article, or claim that may be made by its manufacturer, is not guaranteed or endorsed by the publisher.
