# Supplementary material for: Differential H. pylori-Induced MAPK Responses Regulate Lewis Antigen Expression and Colonization Density on Gastric Epithelial Cells Between Children and Adults
Source: Front Immunol. 2022 Mar 8;13:849512. doi: 10.3389/fimmu.2022.849512 (PMC8957798; doi:10.3389/fimmu.2022.849512)
Supplement: Supplementary file 1 [file DataSheet_1.pdf]

Supplement Fig. 1. Scores of immunohistochemistry stains for MAPK.

p- p38	p- JNK	p- ERK
SE, LP 1<br>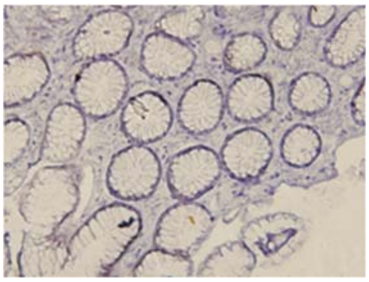	SE, LP 0<br>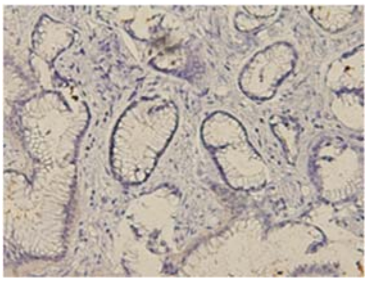	SE, LP 1<br>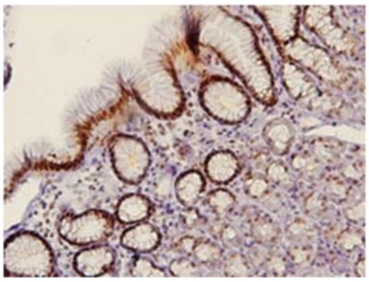
SE, LP4<br>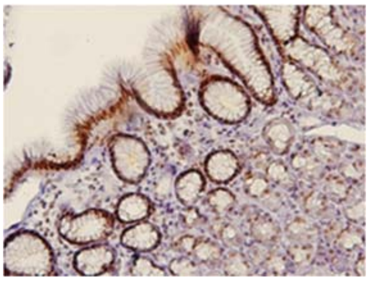	SE, LP4<br>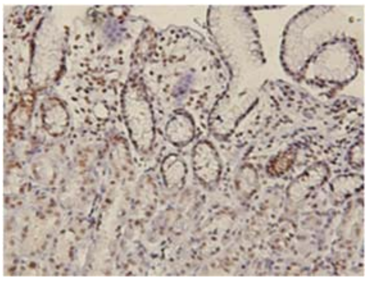	SE, LP 3, 2<br>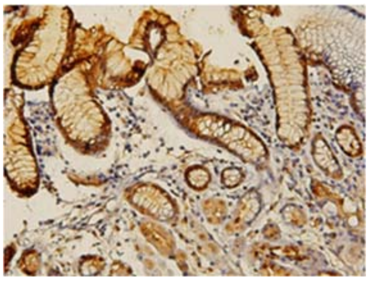
DG 1<br>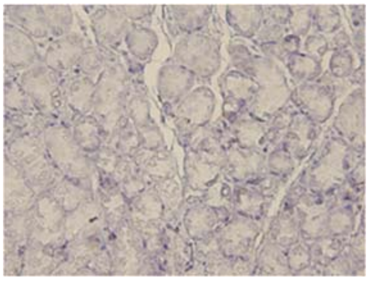	DG 0<br>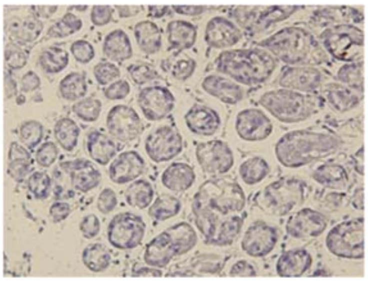	DG 1<br>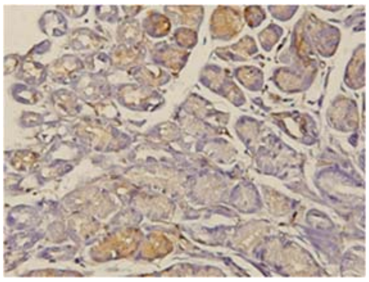
DG 4<br>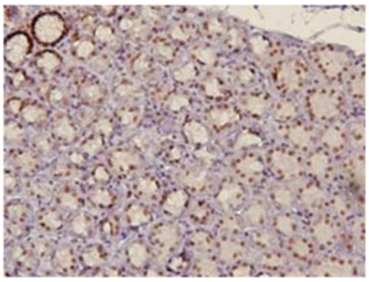	DG 4<br>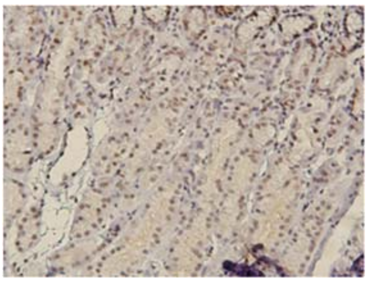	DG 2<br>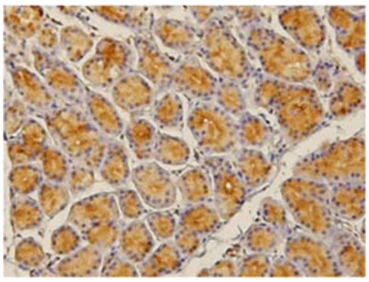

MAPK expression was analyzed in the superficial epithelium cells (SE), in the deep glandular cells (DG), and in the lamina propria mononuclear cells (LP) in nucleus and in cytoplasm. Expression of p-p38, p-JNK, and p-ERK was graded as: 0, negative or less than 1% of stained cells; 1, 1% to 25% of stained cells; 2, 25% to 50%; 3, 50% to 75% of stained cells, and 4, 75% to 100% of stained cells.
